# Photophysical Characterization and In Vitro Evaluation of α-Mangostin-Loaded HDL Mimetic Nano-Complex in LN-229 Glioblastoma Spheroid Model

**DOI:** 10.3390/ijms25137378

**Published:** 2024-07-05

**Authors:** Ammar Kapic, Nirupama Sabnis, Akpedje S. Dossou, Jose Chavez, Luca Ceresa, Zygmunt Gryczynski, Rafal Fudala, Rob Dickerman, Bruce A. Bunnell, Andras G. Lacko

**Affiliations:** 1 Lipoprotein Drug Delivery Research Laboratory, Department of Microbiology, Immunology & Genetics, University of North Texas Health Science Center, Fort Worth, TX 76107, USA; ammarkapic@my.unthsc.edu (A.K.); akpedjedossou@my.unthsc.edu (A.S.D.); bruce.bunnell@unthsc.edu (B.A.B.); 2 College of Science and Engineering, Texas Christian University, Fort Worth, TX 76109, USA; 3 Department of Spine Surgery, Neurological and Spine Surgeon, 5575 Frisco Square Blvd, Frisco, TX 75093, USA

**Keywords:** α-mangostin, rHDL, GBM, LN-229, 3D spheroid

## Abstract

Cytotoxic activity has been reported for the xanthone α-mangostin (AMN) against Glioblastoma multiforme (GBM), an aggressive malignant brain cancer with a poor prognosis. Recognizing that AMN’s high degree of hydrophobicity is likely to limit its systemic administration, we formulated AMN using reconstituted high-density lipoprotein (rHDL) nanoparticles. The photophysical characteristics of the formulation, including fluorescence lifetime and steady-state anisotropy, indicated that AMN was successfully incorporated into the rHDL nanoparticles. To our knowledge, this is the first report on the fluorescent characteristics of AMN with an HDL-based drug carrier. Cytotoxicity studies in a 2D culture and 3D spheroid model of LN-229 GBM cells and normal human astrocytes showed an enhanced therapeutic index with the rHDL-AMN formulation compared to the unincorporated AMN and Temozolomide, a standard GBM chemotherapy agent. Furthermore, treatment with the rHDL-AMN facilitated a dose-dependent upregulation of autophagy and reactive oxygen species generation to a greater extent in LN-229 cells compared to astrocytes, indicating the reduced off-target toxicity of this novel formulation. These studies indicate the potential therapeutic benefits to GBM patients via selective targeting using the rHDL-AMN formulation.

## 1. Introduction

Glioblastoma multiforme (GBM) is an aggressive and essentially incurable glioma that accounts for nearly half of all central nervous system cancers [1]. Life expectancy is often as low as 8 to 24 months due to the location of the tumor and severely limited chemotherapeutic options [2]. A combination of surgical resection, radiation, and high dosages of the chemotherapeutic agent temozolomide (TMZ) have remained the standard management tools for nearly two decades [3,4]. Despite these treatment options, survival beyond two years remains limited at 6.7% [5]. Many treatment challenges contribute to the poor prognosis of the disease. For instance, GBM tumors quickly develop TMZ resistance during the treatment cycle, with extended usage showing no tendency toward improved prognosis [6]. Also, the prolonged use and high dosages of TMZ can induce systemic toxicity in GBM patients [7]. Therefore, there is a dire need to develop alternative and effective therapies, including chemotherapies, for GBM. 

Native to Southeast Asia, the mangosteen (*Garcinia mangostana* L.) fruit is often used in traditional medicines to treat several ailments, including microbial and cardiovascular diseases [8]. These health benefits have earned the increasing popularity of the fruit and have been chiefly attributed to the prenylated xanthone compounds densely packed in the fruit’s rind [9,10]. Among the xanthones associated with the mangosteen fruit 1, 3, 6-trihydroxy-7-methoxy-2,8-bis(3-methylbut-2-enyl)-xanthene-9-one or α-mangostin (AMN) has particularly displayed a robust anticancer activity against cancer cell lines. Regarding GBM, in vitro studies have shown that AMN treatment induced cell death and reduced proliferation and metastatic behavior [11,12]. Mechanistic studies have shown that AMN promotes autophagic cell death and reactive oxygen species (ROS) in cancer cells [13,14].

Evidence of the tumor-selective inhibitory effects exhibited by AMN was initially observed in human leukemia cells that induced apoptosis in the leukemia cell line HL-60; at the same time, normal peripheral lymphocytes were unaffected [15,16]. More recently, AMN treatments in other cancer cell models have revealed additional potential targets, including the mammalian target of rapamycin complex 1 (mTORC1) and cyclin-dependent kinase 4 (CDK4) inhibition, respectively, while exhibiting significantly reduced toxicity towards normal tissue and cells [17,18,19]. Furthermore, AMN treatment induced autophagic cell death in GBM8401 and DBTRG-05MG cell lines through the increased phosphorylation of liver kinase B1 (LKB1), leading to the subsequent activation of AMP Kinase (AMPK) [20]. The increased activation of AMPK has been suggested to play a tumor-suppressive role in glioma and may thus promote autophagic cell death. 

These promising anticancer properties make AMN a potentially effective candidate as a chemotherapeutic agent, even though its hydrophobicity may limit its clinical application. Because AMN is highly hydrophobic with an XlogP3 of 6.27 (far from the ideal range of 1.5–2.7 for solubility in the aqueous environment of the systemic circulation), it will likely require a drug transport system to allow its delivery to the therapeutic target [21]. In addition, its hydrophobic property does not facilitate AMN’s systemic distribution despite possessing a favorable blood–brain barrier (BBB) penetration potential of −0.05 [22,23,24,25]. Furthermore, oral dosages are poorly absorbed, resulting in inadequate bioavailability in relevant target organs [25,26,27], and may induce off-target effects [28,29]. 

Therefore, efficient transport of AMN in therapeutic doses through the systemic circulation likely requires a robust drug delivery system to deliver the drug to cancerous tissues, including in the brain. Lipoprotein-based drug delivery systems answer the call here as they have shown promising results in encapsulating and delivering pharmaceutical payloads, including hydrophobic drugs, imaging agents, and nucleic acids for cancer therapeutics [30,31]. Our group and others have explored the effectiveness of drug delivery via reconstituted HDL nanoparticles (rHDL NPs) for targeting different cancer and immune cells. These studies have shown a positive correlation between the cellular expression of the scavenger receptor class B type 1 (SR-B1), a major gateway for the intracellular uptake of exogenous cholesterol and other hydrophobic cargo from HDL NPs expression, and cellular sensitivity to rHDL NPs-mediated drug delivery [32,33,34,35,36]. Exogenous cholesterol sustains the rapid growth and malignancy of GBM [37]. With this dysregulation of cholesterol metabolism, the upregulation of lipoprotein receptors, including SR-B1, can be observed in GBM cells, especially LN-229 cells [36,38,39]. Since HDL NPs can cross the BBB [40,41], the rHDL NPs platform may be an appropriate vehicle to selectively deliver AMN to high SR-B1-expressing GBM tumors while reducing off-target exposure to healthy brain tissue.

In the present study, we engineered stable, non-leaky HDL NPs nanocomplexes loaded with AMN to form rHDL-AMN NPs. We first reported AMN’s fluorescent properties, which enabled us to probe into the interaction of AMN with the rHDL NPs. The free AMN and rHDL NPs’ photophysical properties demonstrate the drug’s encapsulation within the rHDL NPs. Furthermore, rHDL-AMN NPs displayed enhanced cytotoxic activity, autophagy, and ROS inducement compared to the free AMN and TMZ in LN-229 cells, reducing their migratory potential. Of note, formulating AMN with the rHDL NPs protected astrocytes from the unbiased cytotoxic effect of the free AMN, suggesting a superior safety profile and therapeutic potential of the rHDL-AMN NPs compared to the free AMN or TMZ in GBM in vivo models. 

## 2. Results 

### 2.1. Absorbance and Fluorescence Properties of Free AMN

**Figure 1:**
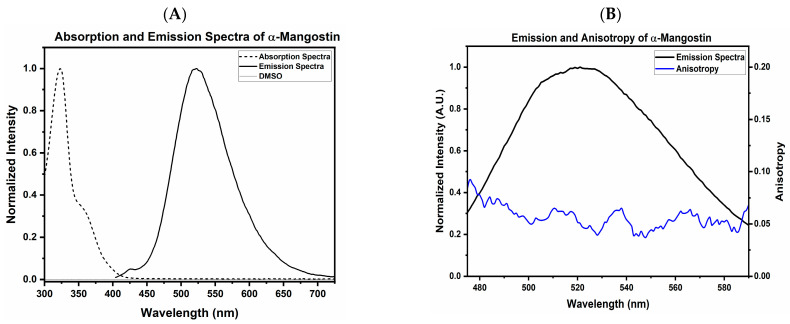
Fluorescence characterization of α-mangostin (AMN). (**A**): Excitation and emission spectra of AMN. (**B**): Anisotropy (blue line) overlaid on top of the emission spectra. The experiment was repeated at least 3 times.

**Figure 2:**
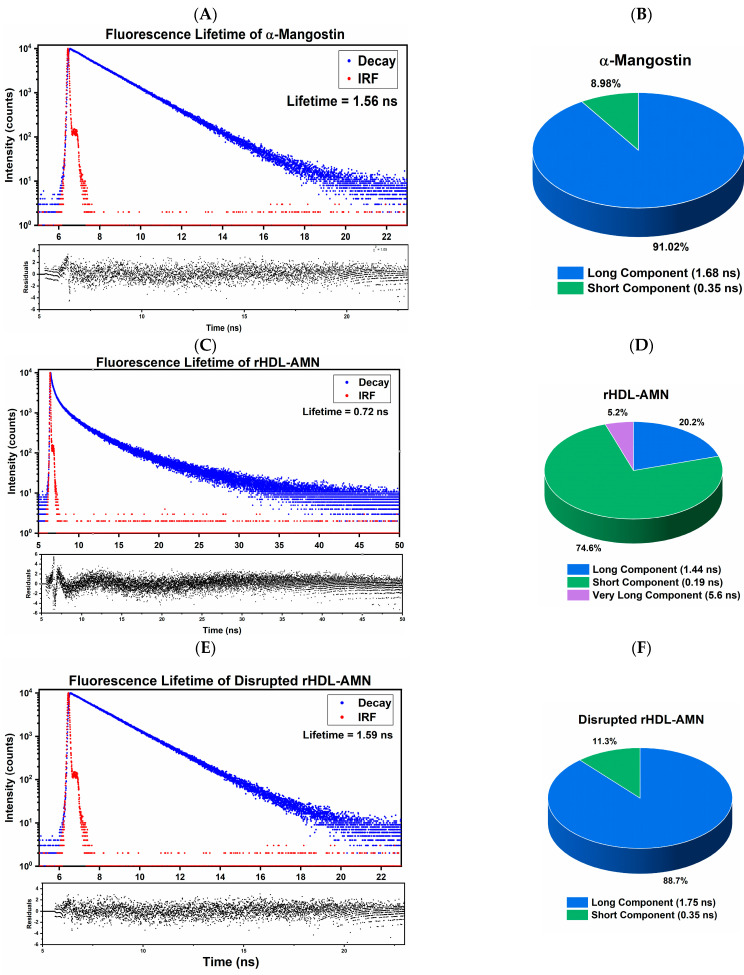
Fluorescence lifetime measurements (FLT) of AMN formulations. (**A**) FLT curve of free AMN; (**B**) The components of the FLT curve of free AMN; (**C**) FLT curve of rHDL-AMN NPs in 1X PBS; (**D**) The components of the FLT curve of rHDL-AMN NPs; (**E**) FLT curve of the disrupted of rHDL-AMN NPs in DMSO; and (**F**) The FLT components of the DMSO-disrupted rHDL-AMN NPs. The experiment was repeated at least 3 times.

The absorbance spectrum of AMN extended from 300 to 405 nm with a maximum of 320 nm, and a bump was observed at 350 nm. Next, using 375 nm as the excitation wavelength, the emission spectrum was measured from 450 to 650 nm, presenting a maximum of 521 nm (Figure 1A). These two spectra revealed a significant Stokes shift of 200 nm between absorption and emission maxima with minimal spectral overlap. The steady-state anisotropy was calculated using the polarized emission components of the AMN in DMSO, which is presented in Figure 1B. The calculated anisotropy is a low-presenting value of ~0.06 in the 521 nm range (Figure 1B), suggesting the high mobility of the free dye. The anisotropy calculated was 0.06 at 521 nm (Figure 1B). Finally, the measured fluorescence intensity decay is presented in Figure 2A. The fit of intensity decay yields two lifetime components, a dominant component of 1.68 ns (91%) and a short component of 0.350 ns (9%) (Figure 2A,B). The calculated amplitude averaged lifetime was 1.56 ns. 

### 2.2. Physical Characterization of Nanoparticles

Lipid-based nanocarriers are characterized by several physical features that influence their in vitro and in vivo behavior and their stability, safety, and effectiveness. These include the average particle size/diameter, the polydispersity index (PDI), which indicates how well-suited they are to the size distribution, and zeta potential (ZP), which determines the probability of aggregation of the formulation. These factors also determine whether or not the formulations are appropriate for a given drug administration route [42]. For nanocarrier formulations to be used successfully in clinical settings, controlling and confirming these parameters are crucial. The physical characterization of rHDL-AMN and empty rHDL formulations is presented in Table 1. Based on the number distribution, the particle diameters for the AMN and empty rHDL NPs were, on average, 40 nm with some variation. The zeta potential was demonstrated to be −24.24 and −29.47 mV, respectively, for the empty rHDL and rHDL-AMN NPs, respectively. Furthermore, the PDI was observed to be 0.2. The formulation was observed to be clear without any precipitation or aggregation. These results suggest that the preparation of rHDL-AMN NPs was relatively homogenous and stable.

### 2.3. AMN Is Associated with the rHDL NPs

The encapsulation efficiency of AMN was 44% using an initial 0.5 mg/mL AMN concentration. Comparison of the steady-state anisotropy (r) value of a fluorophore in its free form to a fluorophore associated with another material can reveal an interaction between AMN and the constituents of the HDL carrier [36,43]. The r value measured for the free AMN (0.06) significantly increases to 0.32 ± 0.05 for the AMN in the rHDL-AMN NPs. This may indicate a significant immobilization of the fluorophore when encapsulated. Also, changes in the FLT for the AMN in the rHDL-AMN NPs, if any, could reveal additional evidence to suggest the encapsulation of AMN in the rHDL-AMN NPs. Samples of the rHDL-AMN and empty rHDL were divided by volume and lyophilized. The amplitude average FLT of AMN decreased from the 1.56 ns recorded for the free AMN (Figure 2A,B) to 0.72 ns in the rHDL-AMN NPs. Analysis of the intensity decay curve of AMN in the rHDL-AMN NPs reveals three lifetime components of 0.190 ns, 1.44 ns, and 5.62 ns. The new long lifetime component of 5.62 ns observed in the rHDL-AMN NPs’ intensity decay is possibly a result of the stabilized interaction of a small AMN fraction that interacts with the rHDL lipoprotein shell. The short component (shorter than the short lifetime component measured for free AMN) that highly increased in fraction (Figure 2C,D) probably reflects the self-quenching of the dye that is densely packed in the NPs (proving an efficient loading). 

The second rHDL-AMN sample was re-suspended in DMSO to destabilize and disrupt the structure of the rHDL, causing the drug’s release. The amplitude average FLT of the DMSO-disrupted rHDL-AMN NPs was comparable to that of the free AMN (Figure 2E,F). Clearly, the FLT components and fractions returned to those measured for free AMN in DMSO. Also, the measured anisotropy of the disrupted samples dropped verified that AMN was unbound (Table 2). Finally, particles were suspended in 1X PBS and stored at 4 °C in a refrigerator for 5 weeks. Table 3 summarizes the change in physical properties of the rHDL-AMN NPs from Day 0 until after 5 weeks. The particles were repassed through a 0.22 µM filter, and the particles were re-analyzed. These results indicate that the AMN is associated with rHDL NPs. Hence, the rHDL-AMN NPs were successfully assembled. 

### 2.4. The rHDL-AMN NPs Are Cytotoxic to the LN-229 Cells and More Protective to Astrocytes than Both the Free AMN and TMZ

**Figure 3:**
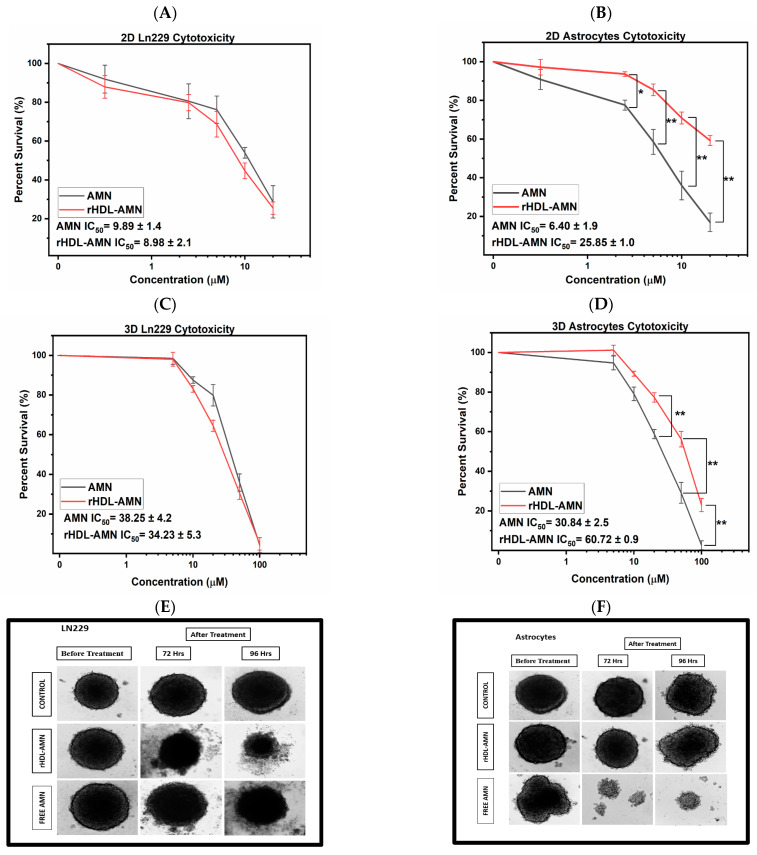

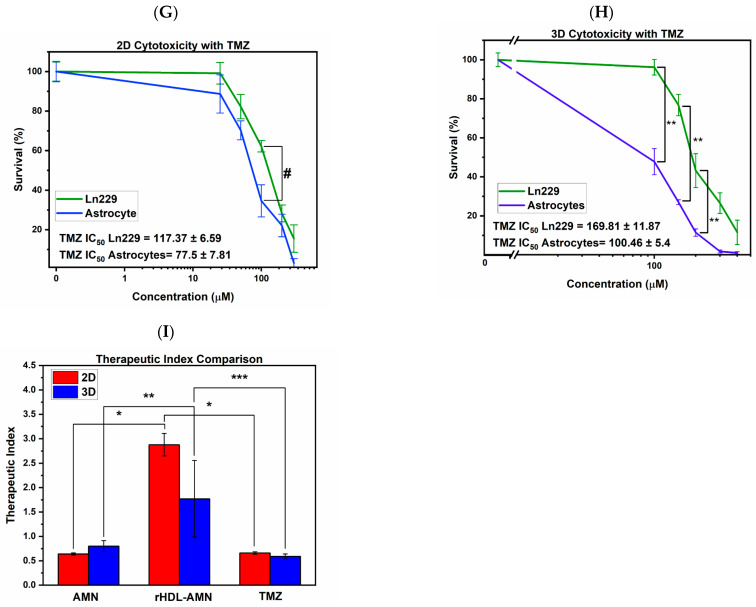
Cytotoxic effect of the free AMN, rHDL-AMN NPs, and TMZ on LN-229 GBM cells and astrocytes. (**A**,**B**) Percent survival featuring free AMN and rHDL-AMN NPs in 2D models of LN-229 and astrocytes, respectively. Statistical significance was conducted using a 2-way ANOVA with a post-hoc Tukey test (* *p* Value = 0.0224, ** *p* value = <0.0001). (**C**,**D**) Percent survival featuring free AMN and rHDL-AMN NPs in 3D models of LN-229 and astrocytes, respectively. Statistical significance was conducted using a 2-way ANOVA with a post-hoc Tukey test (** *p* value = <0.0001). (**E**) Representative images of LN-229 spheroids treated with free AMN or rHDL-AMN NPs. (**F**) Representative images of astrocyte spheroids treated with free AMN or rHDL-AMN. (**G**,**H**) Percent survival of TMZ in the 2D and 3D models conducted using LN-229 cells and astrocytes. Statistical significance was conducted using a 2-way ANOVA with a post-hoc Tukey test (# *p* values = 1.25 × 10^−4^, ** *p* value = <0.0001). (**I**) Therapeutic indices of free AMN, rHDL-AMN, and TMZ with LN-229 cells and astrocytes under given conditions. Statistical significance was conducted using 1-way ANOVA (* *p* values < 0.0001, ** *p* value = 3.68 × 10^−4^, *** *p* value = 2.217 × 10^−4^). Measurements are the average of 3 independent experiments.

The results above demonstrate that AMN was successfully entrapped in the rHDL NPs. However, it is necessary to ensure that the rHDL NPs can complete the intracellular delivery of AMN and that the AMN has retained its cytotoxic activity. Treatment with both the free AMN and the rHDL-AMN (concentration-matched with the free AMN) exhibited a dose-dependent response in both LN-229 cells and astrocytes. After 48 h of treatment in the 2D cell-culture setting, the IC_50_ values calculated for LN-229 in the 2D model with rHDL-AMN NPs formulations and for the free AMN were 8.98 ± 2.08 µM and 9.98 ± 1.4 µM, respectively, suggesting similar cytotoxic capabilities between the free drug and the rHDL-AMN NPs to the GBM cell line. The empty rHDL was used as a control. In contrast, the IC_50_ value of rHDL-AMN formulations was higher (25.8 ± 1.1 µM) than that of the free AMN (6.4 ± 2.4 µM), respectively (Figure 3A,B).

Similar cytotoxic performances of rHDL-AMN NPs and free AMN were observed in the 3D cell-culture setting. Astrocyte spheroids were considerably more affected by free AMN than the rHDL-AMN NPs formulation. Conversely, the encapsulation of AMN within the rHDL NPs did not diminish its cytotoxic effect against the LN-229 cells, thus maintaining its anticancer effects while exhibiting reduced toxicity to the astrocytes (Figure 3C). The IC_50_ values in the LN-229 spheroids were 34.23 ± 6.5 µM and 38.25 ± 4.2 µM with rHDL-AMN NPs and free AMN, respectively, whereas in astrocytes the IC_50_ values were 60.72 ± 1.1 µM, respectively, after 96 h of treatment (Figure 3D). The representative images of LN-229 cells and astrocytes in a 3D model treated with rHDL-AMN and free AMN incubated at 72 and 96 h indicate that astrocytes were well-protected for up to 96 h when treated with rHDL-AMN.

In contrast, free AMN showed the disintegration of spheroids even at 72 h (Figure 3E,F). The effect of TMZ on LN-229 cells and astrocytes in 2D and 3D models demonstrates 1.5- and 1.7-times higher IC_50_ values for LN-229 cells, suggesting that TMZ represented safety issues (Figure 3G,H). The astrocytes showed that the therapeutic index of the rHDL-AMN formulation was 4.5- and 4.3-times higher than that of the free AMN and TMZ formulations in 2D. Meanwhile, the rHDL-AMN NPs were 2.2- and 3-times higher in the 3D spheroid model (Figure 3I). Together, these IC_50_ values and therapeutic indices results demonstrate that the rHDL-AMN NPs are more protective of the astrocytes than the free AMN and TMZ.

### 2.5. The rHDL NPs Can Deliver Their Payload to LN-229 Cells

**Figure 4:**
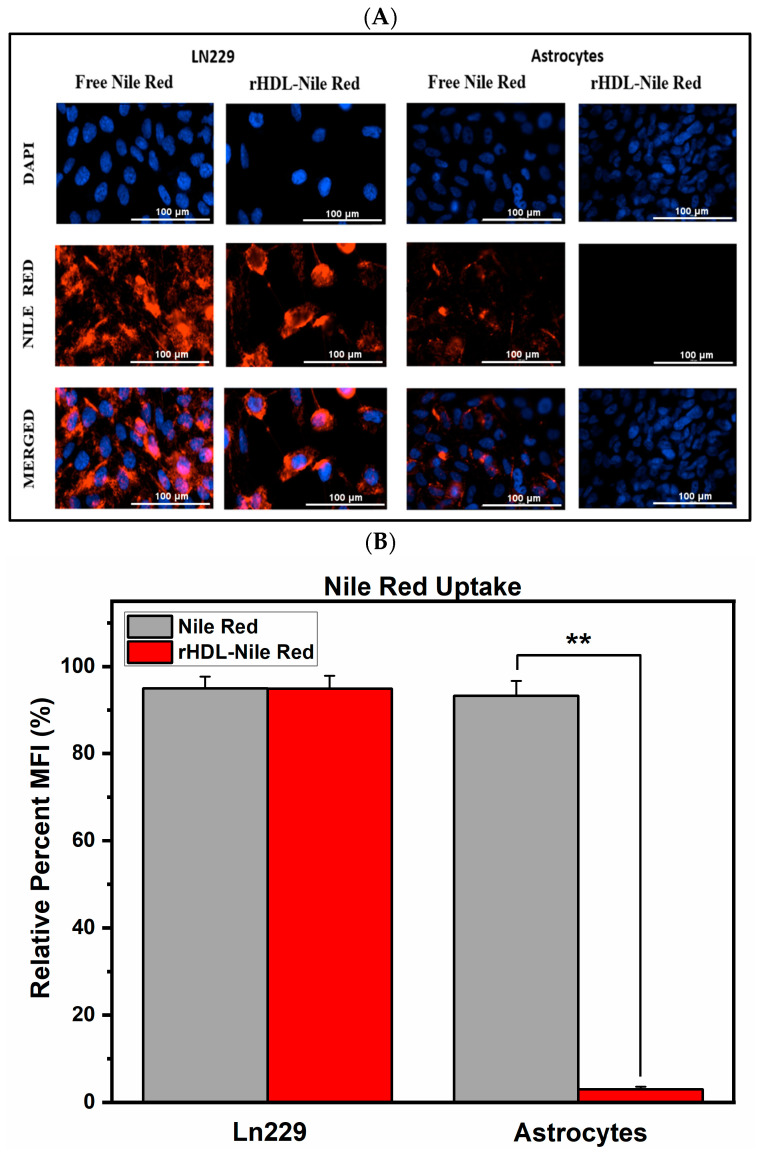
Payload uptake of cargo via rHDL using Nile Red (40× magnification). (**A**) Representative images of the NR uptake in the LN-229 and astrocyte cells incubated with the free NR and rHDL-NR NPs at 1 µM concentration for 1 h at 37 °C; (**B**) Comparison of average NR MFI of the LN-229 and astrocyte cells incubated with the free NR and rHDL-NR NPs. The images and data are representative of three independent experiments and the data are graphed as the mean ± SD. (one-way ANOVA with a post-hoc Tukey test ** *p* value ≤ 0.001).

To visualize the payload uptake mediated by the rHDL NPs in LN-229 cells and astrocytes, we prepared a formulation of rHDL with Nile red (NR), a lipophilic dye, instead of AMN as a proof of concept, since the fluorescence of AMN inside the nanocomplex was very weak. The unloading of the NR into the LN-229 and astrocytes was compared between the rHDL-NR and the free dye and measured as relative percent mean fluorescence intensity (MFI). The cargo uptake via rHDL was equivalent to free NR after 1 h post-treatment in LN-229 cells (>94%); however, in astrocytes, the uptake of the payload was negligible (<3%) with the rHDL-NR formulation, whereas with free NR the uptake was 93% (Figure 4A,B).

### 2.6. AMN Delivery via rHDL NPs Induces Autophagy in LN-229 Cells but Protects Astrocytes against AMN-Induced Autophagy

**Figure 5:**
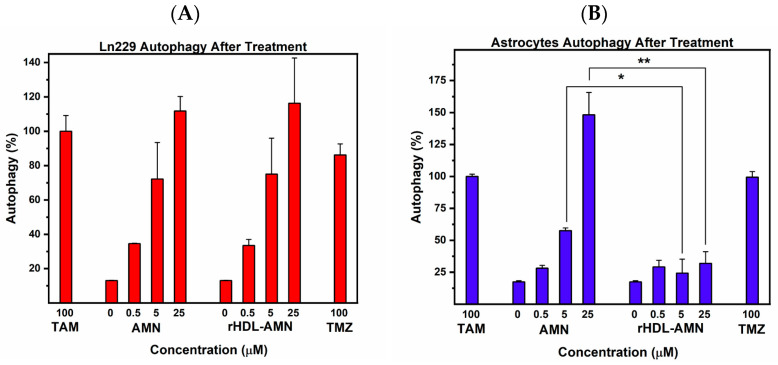
Effect of AMN formulations on autophagy in LN-229 cells and astrocytes. (**A**) LN-229 and (**B**) astrocyte cells were treated with free AMN and rHDL-AMN for 48 h. Using the fluorescence intensity, tamoxifen was used as a positive control to calculate the percentage autophagy in each cell line. Percentage autophagy was calculated considering the positive control as 100%. Statistical significance was conducted using a 2-way ANOVA with a post-hoc Tukey test (* *p* values = 0.0487, ** *p* value = <0.0001). Measurements are the average of 3 independent experiments.

AMN can induce autophagic cell death in glioblastoma cells [44]. To confirm autophagy induction by the rHDL-AMN NPs on glioblastoma cells and the protective effect of using the rHDL NPs on astrocytes, both LN-229 cells and astrocytes were treated with free AMN and rHDL-AMN NPs. Monodansylcadaverine (MDC), which fluoresces once incorporated into multilamellar bodies, was used to detect the presence of autophagic vacuoles. The free AMN and rHDL-AMN NPs exhibited a similar dose-dependent upregulation of autophagy from 34% to 112% in LN-229 cells. However, the astrocytes showed a significant upregulation of autophagy after treatment only with the free AMN, rising from 28.2% to 148%. Unlike the free AMN and TMZ, the rHDL-AMN NP-treated astrocytes demonstrated only an insignificant increase in autophagy with increasing concentrations from 29% to 33% (Figure 5A,B). These results suggest that the rHDL NPs preferentially target the LN-229 cells instead of the astrocytes.

### 2.7. rHDL-AMN NPs Can Delay LN-229 Cell Migration

**Figure 6:**
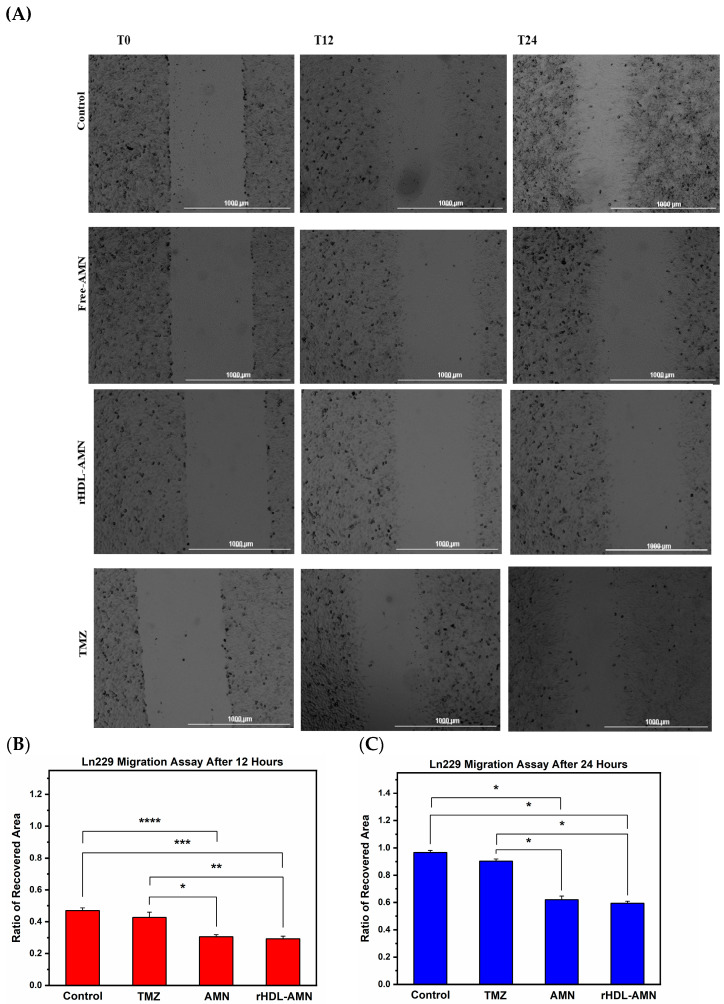
Effect of free AMN, TMZ, and rHDL-AMN NPs at 10 µM drug on the migration of LN-229 cells at 0, 12, and 24 h using a scratch wound assay. (**A**) Representative images (4× magnification) of migration activity after treatment. Scale bars show a length of 1000 micrometers (**B**) and (**C**) Mean ratio of the recovered area at 12 and 24 h respectively using Image J 2.15.0 software. Statistical significance was conducted using a 1-way ANOVA with a post-hoc Tukey test (For (**B**) * *p* values = 0.003, ** *p* value = 9.05 × 10^−4^, *** *p* value 1.15 × 10^−4^, **** *p* value 3.05 × 10^−4^). Statistical significance was conducted using a 1-way ANOVA with a post-hoc Tukey test (For (**C**) * *p* values ≤ 0.0001). Measurements are the average of 3 independent experiments.

It has been demonstrated that AMN can reduce the migratory potential of cancer cells [45]. To confirm that the rHDL-AMN NPs can produce a similar effect in LN-229 cells, we employed the scratch wound-healing assay to measure the cell migratory capability in vitro after treatment with AMN, rHDL-AMN, and TMZ [46]. Whereas the wounds treated with 8 µM of the AMN and rHDL-AMN formulations showed delayed healing, the scars in the untreated control group exhibited considerable cell migration (Figure 6A). In the untreated cells, the recovered area of wound closure ratio was roughly 0.47 at 12 h and nearly 0.97 at 24 h (Figure 6B,C). The ratio of the recovered area of wound closure as a result of free AMN vs. rHDL-AMN treatment decreased by 0.31 and 0.29 after 12 h and 0.82 and 0.79 after 24 h, respectively. The TMZ formulation at an 8 µM concentration showed the recovered area ratio to be 0.43 and 0.90 at 12 and 24 h, respectively.

### 2.8. The rHDL-AMN NPs Induce Higher ROS Levels in LN-229 Cells than in Astrocytes

**Figure 7:**
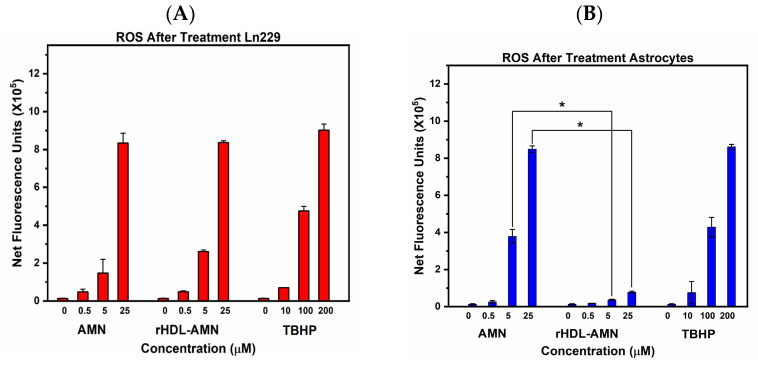
Effect of AMN formulations on reactive oxygen species (ROS activity) in (**A**) LN-229 and (**B**) astrocytes. The ROS activity was measured using the fluorescence of 2′,7′–dichlorofluorescein diacetate (DCFDA) in LN-229 and astrocytes after 24 h treatment with rHDL-AMN and free AMN. Tert-butyl hydroperoxide (TBHP) was used as a positive control. Net fluorescence units were measured at excitation/emission 485 nm/535 nm. Statistical significance was conducted using a 2-way ANOVA with a post-hoc Tukey test (* *p* value ≤ 0.0001). Measurements are the average of 3 independent experiments.

Intracellular levels of ROS can impact tumor progression and the therapeutic response in GBM cells [44,47]. Within the mangostin drug family, γ-mangostin has been reported to increase intracellular ROS [48]. We examined the effect of AMN, rHDL-AMN NPs, and TMZ on LN-229 cells and astrocytes on intracellular ROS. When treated with the free AMN and rHDL-AMN NPs, LN-229 cells showed a dose-dependent increase in ROS activity from 0.5 to 25 µM in concentration (Figure 7A,B). In the astrocytes, only the free AMN increased ROS activity. The rHDL-AMN showed a marginal change in ROS concentrations, suggesting a protective effect.

## 3. Discussion

Since its initial discovery in the 19th century, several biological effects, including anticancer properties, have been reported for AMN [8,9,10,11,12,13,14,15,16,49]. However, poor water solubility and drug bioavailability have limited the use of AMN in therapeutic endeavors. Nanoparticle drug delivery systems have been shown to significantly improve the solubility, tissue infiltration, and pharmacokinetics of drugs and reduce the toxicity of such drugs [50]. In the present study, we formulated AMN with rHDL NPs, and the characteristics of the formulation indicate that the rHDL-AMN NPs were successfully assembled. 

Dynamic light scattering-based measurements showed that the rHDL-AMN NPs have a diameter size well below the recommended size (30–60 nm) for nanoparticles for effective delivery and tumor penetration [43,51,52]. The polydispersity index (PDI) assesses the particle size distribution or homogeneity of the size of nanoparticles in suspension. The PDI can indirectly measure the sample quality of a drug formulation. Poorly stable and heterogeneous preparations exhibit high PDI values closer to one, with homogenous suspensions having values near zero. The PDI for the rHDL-AMN NPs samples meets the recommended threshold of 0.20 and is below the allotted 0.30 for lipid-based nanoparticles [42].

The zeta potential (ZP) probes into the relative stability of the particle in its current solvent. The ZP is measured by the charge difference between the particle’s surface and the solvent [53]. Particles that exhibit a ZP close to 0 mV have a more significant tendency to aggregate due to the lack of repulsive forces on the surface of the particles. Conversely, with ZP values away from zero, there is a reduced likelihood of aggregation of the nanoparticles. Our formulations were reported to have ZP < −24; therefore, they are expected to have a lower tendency for aggregation. The low PDI values suggest that the formulation scheme is consistent in the particle assembly, reflected in the low variance in the hydrodynamic diameter distribution and the zeta potential. 

While quantification is possible for drugs in their nanoparticle formulations, drugs featuring fluorescent properties enable photophysical studies of the drug localization and interaction with its nanoparticle carrier. The fluorescent properties can also facilitate imaging studies to monitor drug delivery and release and determine cellular sub-localization [54,55,56]. The excitation and emission spectra of AMN show a significant Stokes shift separating the absorbance and fluorescence. A large Stokes shift allows for a large separation between excitation wavelength and emission observation, reducing the leak of excitation scattering and significantly improving measurement sensitivity. Also, a small spectral overlap presented by AMN helps to reduce self-quenching. These properties of AMN drugs allow for a simple utilization of steady-state anisotropy and fluorescence lifetime to confirm AMN encapsulation in the rHDL NPs.

The substantial increase in the anisotropy between the free and encapsulated drug (from 0.05 to 0.32, respectively) confirms the significant immobilization of NPs that reduces the molecular rotation of AMN. This phenomenon is due to the external aqueous environment that pushes the hydrophobic drug (i.e., AMN) toward the particle’s hydrophobic core, forcing AMN packing within the core of the rHDL NP [57]. Anisotropy effectively offers a quick, non-destructive method for evaluating the formulation’s quality without significant sample loss [43,54,56]. The FLT was shortened from 1.56 ns to 0.72 ns once associated with the rHDL NPs. The decrease in the FLT suggests a substantial change in the fluorophore environment, potentially tight drug packing or lipoprotein shell components, and/or potential homo-FRET (non-radiative transfer between two comparable probes that show an intersection of the emission and absorption spectra) leading to a reduction in the FLT [43,56,58]. The shortening of the FLT also contributes to the observed anisotropy increase. When the nanocomplex was disrupted, both the FLT and anisotropy returned to the value expected of the free drug, confirming that the AMN was immobilized within the core of the rHDL NPs. Both changes in the FLT and anisotropy can be used as an additional metric for imaging the interactions of the rHDL-AMN NPs with cells and payload release.

The HDL-based NP drug delivery systems developed in our lab and others have demonstrated their potential for targeted drug delivery via SR-B1 receptors [36,59,60,61,62]. We utilized LN-229 (high SR-B1) and astrocytes (low SR-B1) based on our previous studies by Berney et al. [36] to investigate the biological effectiveness of the rHDL-AMN NP formulation and provide an in vitro proof of concept. In 2D culture, both AMN formulations exhibited dose-dependent cytotoxicity, with slightly less IC_50_ for the rHDL-AMN formulation (8.98 µM) than for free AMN (9.89 µM). The 4-fold increase in the IC_50_ of rHDL-AMN NPs over the free AMN formulation in astrocytes resulted in a 4.5-fold improvement in the therapeutic index of rHDL-AMN NPs. These findings illustrated the protective effects of the rHDL NPs on non-cancer cells and enhanced cancer selectivity, which could lessen the potential off-target toxic effects of AMN.

Furthermore, we reported a 4.4-times improvement in the therapeutic index of the rHDL-AMN NPs compared to temozolomide (TMZ), the current first-line chemotherapy agent for GBM. This difference in the therapeutic index could be attributed to the selective delivery of the payload of the rHDL NPs to the LN-229 cells. To validate the effects seen in the 2D culture, we used the 3D spheroid model of GBM in the present study to close the gap between in vitro and in vivo research. The data obtained with a 3D model corroborates the potential of rHDL-AMN as observed in a 2D setting. The therapeutic index is an essential pharmacological metric for evaluating a medication’s safety and effectiveness. By contrasting the dose necessary to provide therapeutic effects with the dose that results in toxicity, it measures the relative safety of a drug [63]. An in vivo study comparing free AMN, rHDL-AMN NPs, TMZ, and associated controls would be important to elucidate the distribution, safety, and importance of SR-B1 in the payload delivery and the therapeutic effectiveness of the rHDL-AMN NPs.

The uptake experiment with NR was designed to show the unbiased uptake of a free payload by the LN-229 cells and astrocytes as opposed to the differential uptake of the payload when it is formulated with the rHDL NPs in astrocytes and LN-229 cells. As evidenced by the in vitro cytotoxicity studies, LN-229 cells are more affected by the rHDL-AMN NPs than the astrocytes are, and our goal was to have a visual representation of this phenomenon mediated by the rHDL NPs with another type of payload. While utilizing the rHDL-AMN NPs for the uptake studies would have been ideal and would have further supported our findings, there are multiple reasons why we selected NR for this study. First, AMN shows very weak fluorescence, and this is not ideal for imaging. In contrast, NR displayed adequate fluoroscopic properties for this particular study. Second, both NR and AMN are highly hydrophobic compounds which have a similar molecular weight range (NR 318.4 g/mol and AMN 410 g/mol). Hence, hydrophobicity and molecular weight were important factors in the interaction of the payload and the rHDL NPs, and NR was considered as an ideal substitute for AMN to study intracellular payload delivery by the rHDL NPs. The uptake studies conducted with NR suggested that the payload delivery was cell-selective, (the astrocytes take up less NR than LN-229 cells do) and could be indicative of protection or less toxicity to the healthy cells when the delivery is mediated via the rHDL NPs. Third, the idea here is to show a proof of concept of payload delivery via rHDL NPs, which we and others have previously elucidated to be SR-B1-mediated [33,35,62]. In addition, in our previous work, Berney et al. have shown that LN-229 is a high SR-B1-expressing cell line, whereas astrocytes are low SR-B1 expressors [36]. The cell-selective uptake of NR we observed in our present study could be suggestive of mediation of SR-B1 in payload delivery via the rHDL NPs. Fourth, the cytotoxic and other anticancer activities reported for the rHDL-AMN NPs coupled with the evidence of the association of AMN with the rHDL NPs in the present study indicates that the rHDL NPs were able to deliver AMN to the LN-229 cells producing anticancer activities whereas astrocytes demonstrated a minimal effect.

Autophagy is a complex and critical cellular process that requires the lysosome’s degradation and digestion of intracellular components. This multistep mechanism keeps fragmented organelles, misfolded proteins, and invasive microbes from building up, allowing cells to recycle and mobilize cell components efficiently [64]. The induction of autophagy by AMN has been reported in other cancer studies, such as breast and osteosarcoma [13,65]. The data shows that rHDL-AMN and free AMN have a dose-dependent autophagic effect on the GBM LN-229 cells. However, only the rHDL-AMN had a protective effect and did not induce autophagy in the astrocytes. These findings corroborate the cancer-targeting effect of the rHDL AMN NPs rHDL and its potential to reduce the side effects caused by AMN.

Prior research has demonstrated that several cancer cells and lymph node metastases have anti-metastatic characteristics in response to AMN [66,67,68,69]. While numerous cancer-related signal transductions and matrix metalloproteases have been shown to have their expression reduced by AMN, the precise method by which AMN inhibits metastasis is still unknown [67,70,71,72]. However, Nalla et al. have shown that AMN’s anti-migratory and anti-proliferative effects in breast cancer cells may be through STAT3 inhibition [45]. The greatest obstacle to current curative therapy for GBM tumors has been identified as their tendency toward exceedingly aggressive invasion of other regions of the brain [73]. Utilizing the wound-healing assay, we demonstrated that the free AMN and rHDL-AMN NPs exhibited delayed migration effects against GBM compared to the control and TMZ. 

Finally, an overabundance of ROS and disruption of antioxidant homeostasis are hallmarks of oxidative stress that can promote cell death. The present work investigated the effect of rHDL-AMN NPs on ROS modulations on the GBM cells and astrocytes. The LN-229 and astrocytes demonstrated a dose-dependent elevation of ROS in response to free AMN. However, the rHDL-AMN NPs did not induce elevated ROS in the astrocytes due to the cancer selectivity of the rHDL-AMN NPs. As we and others have shown in other cancer models, the protective effect observed on astrocytes (normal cells) by rHDL-AMN NPs is likely due to its receptor-mediated delivery of the payload [36,74,75].

## 4. Materials and Methods

### 4.1. Materials

The AMN was obtained from Selleckchem (Catalog Number: S3804), Radnor, PA, USA. Egg yolk phosphatidylcholine (EYPC), free cholesterol, sodium cholate, uranyl acetate, and dimethyl sulfoxide (DMSO) were purchased from Sigma-Aldrich, St. Louise, MO, USA. Apolipoprotein A1 (ApoA1) was received from Cerenis Therapeutics, Labege, France. Quartz cuvettes with dimensions of 0.1 cm were obtained from Thor Labs, Newton, NJ, USA. Dialysis cassettes, a 20,000 MWCO Slide-A-Lyzer, and fluorescence filters were obtained from Edmund Industrial Optics Barrington, NJ, USA. The manual polarizer was obtained from Varian Palo Alto, CA, USA. Malvern Zetasizer 1070 Capillary Cells were purchased from Malvern (Malvern, UK).

### 4.2. Preparation of rHDL-AMN NPs

**Figure 8:**
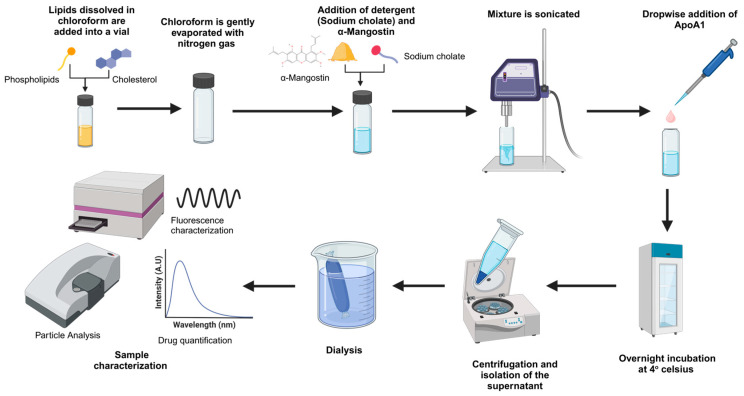
A schematic representation of the rHDL-AMN nanoparticle synthesis protocol and characterization process.

A previously established rHDL protocol was modified for formulating the rHDL-AMN NPs [36]. The lipid portion of the rHDL was prepared first by mixing egg yolk phosphatidylcholine (EYPC) and free cholesterol (FC) dissolved in chloroform. Next, the lipid mixture was evaporated under nitrogen gas at room temperature to remove the chloroform. To this mixture, sodium cholate was added, and the solution was vortexed until no visible residue was present on the vial walls. To this mixture, the required amount of AMN from a stock of AMN dissolved in DMSO was added directly to the solubilized lipid mixture. Figure 8 schematically represents the synthesis process of rHDL-AMN. 

The mixture was sonicated at an amplitude of 80 in continuous mode using the sonicator (Qsonica, L.L.C., Newton, CT, USA) and allowed to rest on ice for 2 min. The sonication step was repeated five times. Once the lipids and AMN were settled, the solution was continuously stirred. Finally, ApoA1 was added dropwise to reduce aggregation and enhance nanoparticle formation. The samples were then incubated overnight at 4 °C on a nutator shaker. After incubation, the particles were purified utilizing dialysis. The samples were loaded by syringe into dialysis cassettes (20,000 MW), and the cassette was kept in 1X PBS solution for 48 h with buffer change at least three times. After purification, the samples were centrifuged at 12,000 RPM for 1 h at 4 °C. The samples were then filtered using 0.22 µm cellulose syringe filters and were stored at 4 °C until characterization. Empty NPs lacking the drug were also formulated simultaneously for each batch to assess the batch quality and as a reference blank for characterization.

### 4.3. Characterization of rHDL-AMN NPs

The physical characteristics of rHDL-AMN were determined with dynamic light scattering (DLS) using the Malvern Zetasizer Ultra Particle Analyze (Malvern, United Kingdom). The samples were diluted to a 1:15 concentration in deionized, filtered water at room temperature. For size and PDI determination, 10 × 10 × 23 mm plastic cuvettes were used. For the zeta potential measurement, Malvern 1070 folded capillary cartridges were used. The hydrodynamic diameter was reported according to the peak of the number distribution.

## 5. Photophysical Characterization

### 5.1. Spectroscopic Characterization of the Free AMN

To uncover the photophysical characteristics of the free AMN, aliquots of different concentrations were prepared using DMSO in 0.1 cm quartz cuvettes. All measurements were conducted at room temperature after the drug was fully thawed. First, the absorbance spectrum was measured with DMSO as a blank using the Cary UV-60 spectrophotometer (Agilent Technologies, Santa Clara, CA, USA). Next, the emission spectra were measured using the Cary Eclipse system. Finally, the anisotropy was determined by measuring the polarized intensity of the samples. Equation (1) was used to calculate the steady-state anisotropy (r), where *I_vv_* and *I_vh_* denote the fluorescence intensities measured with vertical excitation and vertical and horizontal observations, respectively. The G-Factor (G) was measured with horizontal excitation and was calculated using Equation (2) [43], where *I_hv_* and *I_hh_* are vertical and horizontal fluorescence intensities measured with horizontal excitation, respectively.

(1) $$r=\frac{I_{vv}-GI_{vh}}{I_{vv}+2GI_{vh}}$$

(2) $$G=\frac{I_{hv}}{I_{hh}}$$

Finally, the fluorescence lifetime was determined using a time-correlated single photon counting (TCSPC) module (Picoharp 300) with a Fluo-Time 300 fluorometer (PicoQuant, Berlin, Germany. The samples were excited using a 375 nm pulsed diode laser (PicoQuant, GmbH), and the observation wavelength was set to 520 nm. The fluorometer was equipped with an ultrafast microchannel plate detector (MCP) from Hamamatsu, Inc (Iwata City, Japan). The fluorescence lifetimes were measured in the magic angle condition, and data were analyzed using the FluoFit 4.0 software from PicoQuant, Inc. (GmbH) and the multi-exponential fitting model. The lifetime decays were fit according to Equation (3) [76].

(3) It=∫−∞tIRF(t′)∑iαie−(t−t′)τi       

Equation (3) represents a deconvolution model where IRF(*t*′) is the Instrument Response Function at *t* = *t*′. The $\alpha_{i}$ term represents the amplitude for the ith intensity decay component at time t_o_, and $\tau_{i}$ represents the lifetime of that component. Equation (4) was used for calculating intensity (<τ>_int_), and Equation (5) was used to calculate amplitude (<τ>_amp_) average decays; the following formulas were used:

(4) $${<\tau >}_{int}=\sum f_{i}\tau_{i}$$

(5) $${<\tau >}_{amp}=\sum \alpha_{i}\tau_{i}$$

(6) $$f_{i}=\frac{\sum \alpha_{i}\tau_{i}}{\sum \alpha_{i}}\;\;\;$$

### 5.2. Entrapment Efficiency and Photophysical Characterization of rHDL-AMN NPs

Absorbance values for AMN at 320 nm were obtained using the Cary UV-60 (Agilent, Santa Clara, CA, USA) spectrophotometer. Serial dilutions of free AMN in DMSO were prepared to create a standard curve to quantify the drug concentration in the rHDL-AMN NPs samples. The rHDL-AMN NPs samples were diluted by 1:10 using 1X PBS and blanked using their solvent. Empty rHDL NPs were used to remove the scattering profile of the particles from the rHDL-AMN NPs measurements to correct the spectra. These absorbance values would then be converted into molar concentration values based on the standard curve. The encapsulation efficiency (EE), shown in Equation (7), was determined by dividing the concentration of AMN in the rHDL-AMN NPs recovered after dialysis by the molar concentration of AMN estimated at the initial multiplied by 100 to give a percent retention.

(7) $$\%EE=\frac{AMN\;concentration\;after\;dialysis}{AMN\;concentration\;recorded\;at\;the\;initial\;addtion\;of\;AMN\;\;}\times 100$$

For fluoroscopic studies on AMN, filters were utilized on the Cary Eclipse system to reduce signal noise, with a 495 long-pass filter placed near the emission detector and a 405 short-pass filter placed at the excitation lamp. Further signal noise reduction to remove the emission intensity of the lipoprotein shell required the empty rHDL NPs. The scattering profiles of the rHDL-AMN NPs were matched accordingly to the empty rHDL NPs. Then, the intensity of the empty particles was removed from the rHDL-AMN NPs. The intensity value at 520 nm was used for calculating the steady-state anisotropy.

The fluorescence lifetime (FLT) measurements of the rHDL samples were performed using the Fluo-Time 300 (PicoQuant, Berlin, Germany), with the same conditions, equipment, and set-up as the free AMN characterization. The intensity decay was calculated using a tail fitting on the Origin software 2022b/2023b. To disrupt the rHDL-AMN NPs, a sample was split by volume into two glass vials. Both samples were lyophilized using the Labconco Freezone 12 Lyophilizer. The samples were placed at −80 degrees overnight and then lyophilized for 24 h. The samples were re-suspended in either deionized water or DMSO before the measurements. After adding the solvent, the samples were vortexed at low speed until no residue was visible on the vial walls before immediately being measured by the Fluo-Time 300.

### 5.3. Cell Culture Conditions 

The glioblastoma cell line LN-229 was obtained from ATCC. Primary human astrocytes were obtained from Dr. Kathleen Borgman lab in UNT Health Science Center using first or second trimester brain tissues from a biorepository at the University of Washington and in full compliance with local, federal, and National Institutes of Health (NIH) ethical guidelines. Written informed consent was obtained from all donors. The isolation and characterization of astrocyte cultures are previously described [77,78,79]. Briefly, the cell lines were grown in DMEM-F12 medium (Gibco Thermo Fisher Scientific, Grand Island, NY, USA) with 10% fetal bovine serum (FBS) and 1% penicillin–streptomycin. The cell cultures were maintained in a humidified atmosphere at 37 °C, 95% air, and 5% CO_2_. The cells were passaged using 0.25% trypsin to detach the cells from the flasks once 80–90% confluency was reached. Spheroid models of the above cell lines were established using an ultra-low cluster, ultra-low attachment round-bottom 96-well plates, costar, Corning Inc. An initial 5000 cells per well were used for all the cell lines. The cells were grown in DMEM-F12 medium containing 10% FBS and 1% penicillin–streptomycin and incubated in the humidified atmosphere at 37 °C, 95% air, and 5% CO_2_ on a shaker with 50 rpm for at least four days until spheroids of the desired size (between 400 and 600 nm in diameter) were formed. 

### 5.4. Cytotoxicity Studies with Free AMN and rHDL-AMN NPs

The cytotoxicity of the formulations was studied using a cell counting kit (CCK-8) from Dojindo Molecular Technologies (Tabaru, Kumamoto, Japan). The test utilizes the property of a compound, WST-8 (2-(2-methoxy-4-nitrophenyl)-3-(4-nitrophenyl)-5-(2,4-disulfophenyl)-2H-tetrazolium, monosodium salt), which produces a water-soluble, orange formazan dye upon bio-reduction by cellular dehydrogenases in the presence of an electron carrier, 1-Methoxy PMS. The cell lines were grown according to the procedures and culturing conditions provided by the ATCC. Cell density (cell count) was determined using the Cell drop Brightfield cell counter (DeNovix Inc., Wilmington, DE, USA). To initiate cell growth, 5000 cells per well were seeded into 96-well microtiter plates and incubated at 37 °C in 5% CO_2_ for 24 h to allow the cells to attach to the plates. The free drug and the rHDL NPs were diluted in DMSO + 2% DMEM-F12 medium and 2% DMEM-F12 medium, respectively, to yield stock solutions of equivalent molar concentrations. Subsequently, aliquots of the stock solutions were added to microtiter plate wells to achieve the required concentrations for cell viability. Control treatments included no treatment, vehicle alone, and control without cells with the same formulations for each concentration. Cells were incubated at 37 °C in 5% CO_2_ for 24–72 h. After incubation, 10 μL of highly water-soluble tetrazolium salt, WST-8 stock solution was added to each well. After 3 h of incubation at 37 °C, the absorbance at 450 nm was measured using a Bio-Tek Cytation 3 image reader (Agilent, Santa Clara, CA, USA). The concentration required to achieve 50% cell growth inhibition (IC_50_) was calculated according to the manufacturer’s instructions. Six replicates were used at each concentration. For studies comparing the cytotoxic effect of free AMN, rHDL-AMN NPs, and TMZ on both LN-229 cells and astrocytes, cell survival was measured by CCK-8 assay after 72 h in a 2D monolayer and 96 h in a 3D spheroid model. After the cytotoxicity studies, the percentage survival was calculated using Equation (8) below, where A_t_ is the net absorbance of the treatments and A_c_ is the net absorbance of the control without the drug. The IC_50_ value was extrapolated from the graph of log concentration vs. percentage survival. For comparison between free AMN, rHDL-AMN, and TMZ, the therapeutic index of the AMN formulations was calculated using Equation (9) [63] based on the IC_50_ values in 2D and 3D models of astrocytes and LN-229 cells.

(8) $$\%\;Survival=\frac{A_{t}}{A_{c}\;\;}\times 100$$

(9) $$Therapeutic\;Index=\frac{{IC50}_{astrocytes}}{{IC50}_{LN-229}\;\;}\;$$

### 5.5. Payload Uptake Studies

For visualization and to confirm cargo uptake from the rHDL NPs by the LN-229 cells and low uptake by astrocytes, rHDL Nile Red (NR) NPs were assembled using the same preparation method as for rHDL-AMN described earlier. NR is quantified via absorbance at 550 nm using a Cary 60 UV–Vis spectrophotometer, Agilent, Santa Clara, CA. LN-229 cells at a 5 × 10^5^ cell density were plated in 35 mm glass-bottom dishes. After the attachment of the cells with overnight incubation, the cells were treated with either free NR (dissolved in DMSO) or the rHDL-NR. The free Nile Red and the rHDL–Nile Red NPs were matched to the same concentration (1 μM NR). After one hour of incubation at 37 °C, the treatments were washed three times with 1X PBS and incubated with 5 μM Hoechst 33342 solution, Thermo Fisher Scientific Inc. (Waltham, MA, USA), in 1X PBS for 15 min, followed by three washes of 1X PBS. The cells were then re-incubated in phenol red-free DMEM-F12 media supplemented with 2% FBS and visualized using the Bio-Tek Cytation 3 imaging reader (Agilent Technologies Inc., Santa Clara, CA, USA). Images were recorded at 4× and 40× magnification using DAPI and Texas Red filters. At least six fields were recorded, and the experiment was repeated thrice. 

### 5.6. Autophagy Assay

The effect of the rHDL-AMN NP formulations on autophagy was determined using Monodansylcadaverine (MDC) (Abcam, Waltham, MA, USA) according to the manufacturer’s suggestions. Briefly, 96-well black culture plates were seeded with LN-229 and astrocytes at 5 × 10^4^ cells/well. Cells were grown overnight at 37 °C with 5% CO_2_ in DMEM-F-12 medium. The next day, the cells were treated with AMN formulations at concentrations ranging from 0 to 25 µM for 48 h. After the treatment, the plates were centrifuged at room temperature for five minutes at 400× *g*. The supernatant medium was aspirated. Then, 100 µL of the cell-based MDC solution, except for the background wells, was added to each well. The plates were incubated for ten minutes at 37 °C. The supernatant was aspirated, and the cells were washed with the assay buffer. The cells were again centrifuged at room temperature for five minutes at 400× *g*. After aspirating the supernatant, 100 µL of phenol red-free DMEM-F-12 medium was added to the wells. The cells were immediately analyzed in the Bio-Tek Cytation 3 image reader(Agilent, Santa Clara, CA, USA),to read the plate using an excitation wavelength of 335 nm and an emission wavelength of 512 nm. A graph of fluorescence intensity vs. concentration of AMN was plotted. The experiment was repeated at least three times.

### 5.7. Wound Healing Assay

LN-229 cells were plated in 12-well plates at a density of 2 × 10^5^ cells using a regular cell-culture medium and cultured for 24 h. After the cells achieved confluence, a wound was created by scratching through the middle of the dish with a 200 mL tip [69]. Cells were gently rinsed twice with the culture medium to remove floating cell debris. The medium alone was added to the control dish; the medium diluted with mangostin at a final concentration of 8 µM was added to the treatment dish. The first image acquisition (t = 0 h) was then performed using a Bio-Tek Cytation 3 image reader. Cells were then cultured for the subsequent image acquisition (t = 12 h and t = 24 h). The data were analyzed by ImageJ software (NIH, Bethesda, MD); the ratio of the recovered area, Ar, which was covered by cells, was calculated in Equation (10), where A_0_ is the scratched area at t = 0 h, and A_covered_ is the area covered cells at different incubation times (t = 12, 24 h). The covered areas were compared using nonparametric analyses of variance followed by the Kruskal–Wallis H test and Steel pairwise comparison test.

(10) $$A_{r}=\frac{A_{covered}}{A_{0}\;\;}$$

The images are taken at 4× magnification with a scale bar of 1000 µm. The experiment was repeated at least 3 times and the area recovered shown indicates the average value. 

### 5.8. Detection of Reactive Oxygen Species (ROS)

The ROS assays were performed using the DCFDA/H2DCFDA-Cellular ROS Assay Kit (Abcam, Waltham, MA, USA). The procedure for adherent cells was followed according to the manufacturer’s protocol for microplate assay. Briefly, the LN-229 and astrocytes cells were seeded on a plate and maintained in DMEM-F12 media in a dark, transparent bottom 96-well microplate with 25,000 cells per well. The cells were allowed to adhere overnight. The cells were then treated with different concentrations of AMN formulations and incubated at 37 °C with 5% CO_2_ for 24 h. The media was removed, and 100 μL/well of 1X buffer was added. After removing the buffer, 100 μL/well of the diluted DCFDA reagent was added. The cells were incubated with the diluted DCFDA solution in the dark for 4 h at 37 °C. After incubation, the DCFDA solution was removed, and the cells were washed with 100 μL/well DMEM medium with 10% FBS without phenol red. Tert-butyl hydroperoxide (TBHP) was used as a positive control. In endpoint mode, plates were immediately measured using a Bio-Tek Cytation 3 fluorescence plate reader (Agilent, Santa Clara, CA, USA) at Ex/Em = 485/535 nm.

### 5.9. Statistics

Unless otherwise noted, every study was carried out in at least three separate experiments with replicates ≥ 3 for each experiment. OriginPro 2022b/2023b software (OriginLab Corp., Northampton, MA, USA) was used to evaluate the data. An unpaired two-tailed Student’s *t*-test was employed for comparisons only involving two groups. When more than two treatment groups were being compared, a one-way or two-way ANOVA with a post-hoc Tukey test was utilized to assess the statistically significant differences in treatment responses. At *p* < 0.05, statistical significance was assessed. The results’ mean plus standard deviation (SD) is displayed for experimental trials, N ≥ 3.

## 6. Conclusions

Alpha-mangostin (AMN), one of the most abundant prenylated xanthones that exists in the pericarp of mangosteen fruit, has been demonstrated as a promising compound that has multiple health benefits including natural anticancer properties. However, its effectiveness can be limited by poor solubility and bioavailability. To address this issue, in the present study, an HDL-mimetic nanocomplex (rHDL) has been investigated as a potential delivery system to enhance the cytotoxicity to cancer cells and improve selectivity against normal cells using spheroid models of Glioblastoma multiforme (GBM), one of the most aggressive and incurable gliomas, and astrocytes, the most abundant glial cells. In this study, rHDL-AMN NPs were developed to take advantage of the hydrophobicity of AMN and enhance its cancer-selective properties. The unique fluorescent properties of AMN allowed the utilization of steady-state anisotropy and fluorescence lifetime to confirm AMN encapsulation in the rHDL NPs. The anisotropy measurements effectively offered a quick, non-destructive tool for evaluating the formulation’s quality without significant sample loss. We demonstrated the enhanced cancer-targeting potential of rHDL-AMN, as astrocytes were significantly less impacted than the GBM cells LN-229 when treated with the rHDL-AMN nanocomplexes. These findings align with other reports assessing the potential benefits of rHDL NPs as therapeutic nanocarriers. It was also shown that the rHDL-AMN formulation was superior to TMZ, a first-line therapy of GBM currently used at lower doses to restrict the migration of GBM cells. Further research is needed to assess the capability of rHDL-AMN NPs to pass the blood–brain barrier to validate their future applicability in the brain delivery of drugs, particularly towards GBM therapeutics. This study still requires confirmation in vivo and selectivity tests in other glial cells.

## Figures and Tables

**Table 1:** Physical characteristics of the rHDL-AMN NPs and empty rHDL NPs *.

Characteristics	Empty rHDL	rHDL-AMN
Diameter size (nm)	40.26 ± 1.28	38.99 ± 9.43
Polydispersity index	0.19 ± 0.01	0.19 ± 0.02
Zeta potential (mV)	−21.49 ± 2.7	−29.47 ± 8.3

* The experiment was repeated at least 3 times (N ≥ 3).

**Table 2:** Anisotropy of disrupted and undisrupted samples of rHDL-AMN NPs.

Samples	r Value
Free AMN	0.06 ± 0.002
rHDL-AMN NPs	0.32 ± 0.006
DMSO-disrupted rHDL-AMN NPs	0.061 ± 0.001

Note: The experiment was repeated at least 3 times and the values represented are average with standard deviation.

**Table 3:** Storage stability of the rHDL-AMN NPs and empty rHDL NPs after 5 weeks with respect to the physical characteristics of the particles.

Empty rHDL			
Incubation Duration	PDI	Particle Diameter (nm)	Zeta Potential (mv)
Day 0	0.19 ± 0.01	41.26 ± 1.28	−21.49 ± 2.7
5 weeks (before filtering)	0.19 ±0.02	38.6 ± 3.5	−22.25 ± 3.4
5 weeks (after filtering)	0.18 ± 0.01	40.84 ± 2.6	−20.84 ± 4.6
**rHDL-AMN**			
Day 0	0.19 ± 0.02	38.33 ± 2.19	−29.47 ± 8.3
5 weeks (before filtering)	0.25 ± 0.04	26.67 ± 2.87	−30.21 ± 2.60
5 weeks (after filtering)	0.19 ± 0.02	35.03 ± 7.03	−24.45 ± 0.88

Note: The experiment was repeated at least 3 times and the values represented are the average with standard deviation.

## Data Availability

The data presented in this study are available on request from the corresponding authors.
